# Supplementary figures and images for: Heterogeneous Network Edge Prediction: A Data Integration Approach to Prioritize Disease-Associated Genes
Source: PLoS Comput Biol. 2015 Jul 9;11(7):e1004259. doi: 10.1371/journal.pcbi.1004259 (PMC4497619; doi:10.1371/journal.pcbi.1004259)

## High Confidence Association:

Primary Gene: HLA-DRB1

Secondary Genes: HLA-DRA, C6orf10

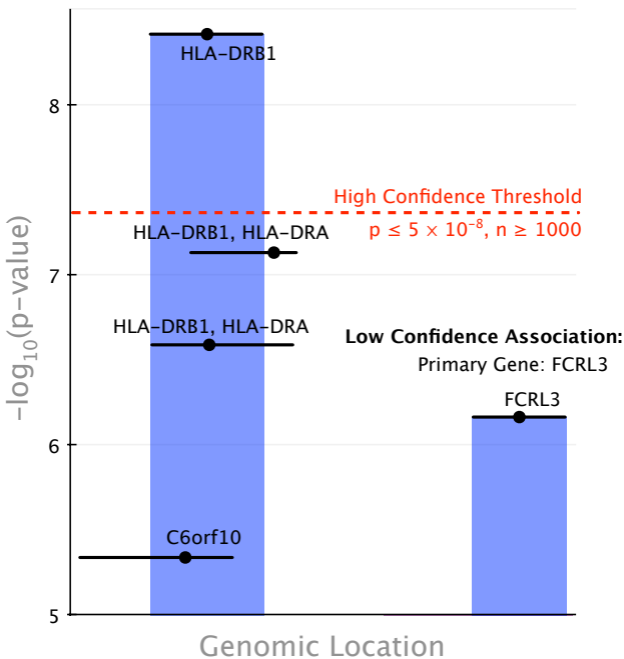

Supplement: S1 Fig — First, associations from the GWAS Catalog are segregated by disease. In this hypothetical example, we show a selection of multiple sclerosis associations. Each association consists of a lead SNP (point) and window (line segment) within which the causal SNP is expected to reside. In order of significance, association windows are overlapped into disease-specific loci (blue bars). For loci identified by multiple studies, the most commonly reported gene is considered primary and the remainder are considered secondary. A loci is classified as high-confidence if any of its associations exceed the threshold shown by the dashed red line. (PDF) [file pcbi.1004259.s001.pdf]

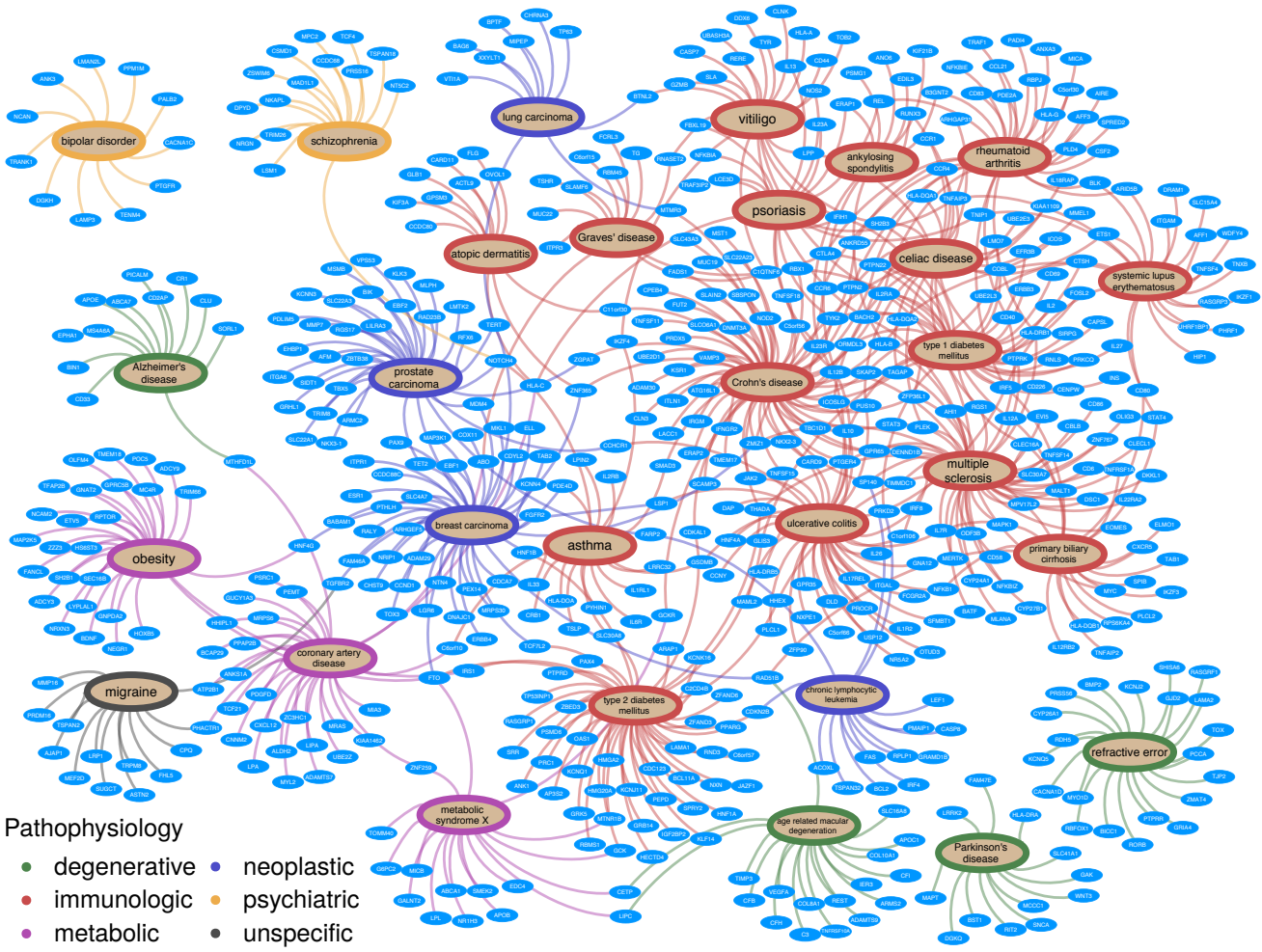

Supplement: S2 Fig — Gene-disease associations were extracted from the GWAS Catalog. Here we show the 698 high-confidence primary associations for the 29 diseases with at least 10 associations. Diseases (large nodes) and their incident edges are colored according to disease pathophysiology. Node positions were manually adjusted for clarity after an initial force-directed layout. The network highlights pervasive pleiotropy as well as the overlap of susceptibility genes among autoimmune diseases. (PDF) [file pcbi.1004259.s002.pdf]

10 × 20 fold CV AUROC

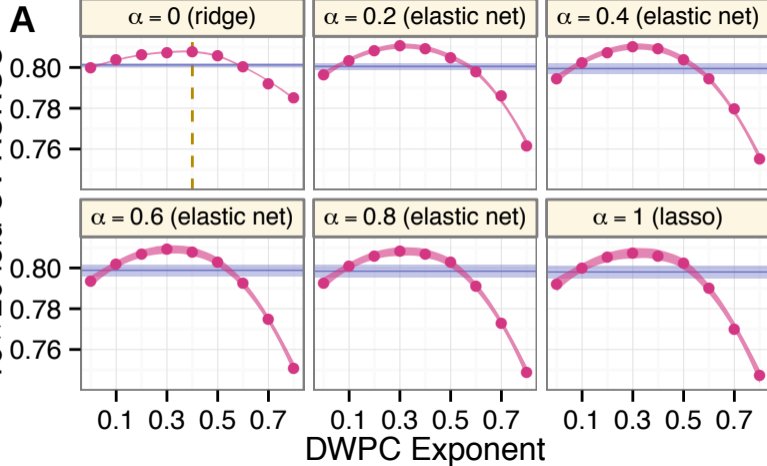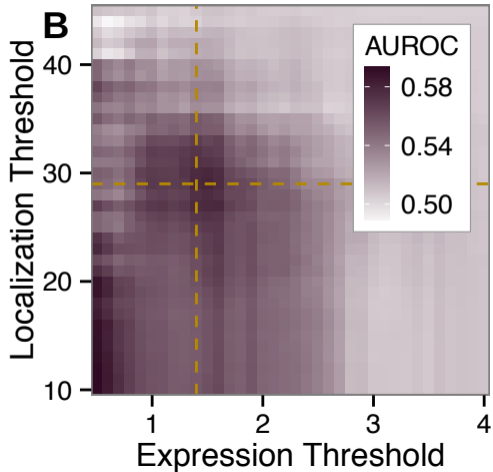

Supplement: S3 Fig — Using the training network, optimal parameter values (yellow dashed lines) were chosen. A) Using average cross-validated AUROC to assess performance, six elastic net mixing parameters were evaluated. For each mixing parameter value α, 10 feature metrics were evaluated: the DWPC for 9 weighting exponents (w, magenta with a 99.99% loess confidence band) and the NPC (violet with a 99.99% confidence interval). The DWPC with w = 0.4 outperformed the NPC, the best metric from previous work, as well as the path count which equals the DWPC when w = 0. Performance variability was minimized when α = 0. B) Edge-inclusion thresholds for two metaedges were jointly optimized. Expression threshold refers to the minimum microarray intensity required for a tissue-specific expression (GeT) edge. Localization threshold refers to the minimum literature co-occurrence score required for a disease localization (TlD) edge. Treating the DWPC (w = 0.4) for the GeTlD metapath as a classifier, the AUROC was calculated at each pairwise threshold combination. The optimal thresholds were chosen as the center of a stable, high-performing, and computationally-feasible section of the solution space. (PDF) [file pcbi.1004259.s003.pdf]

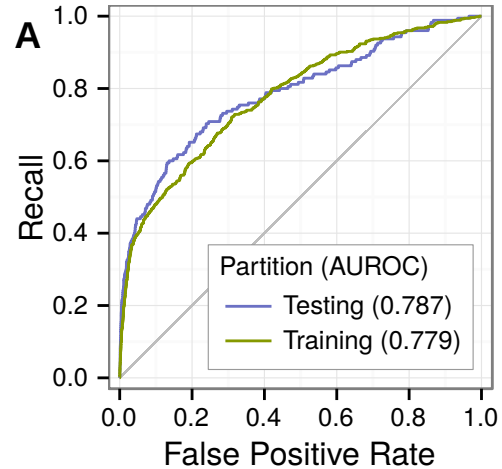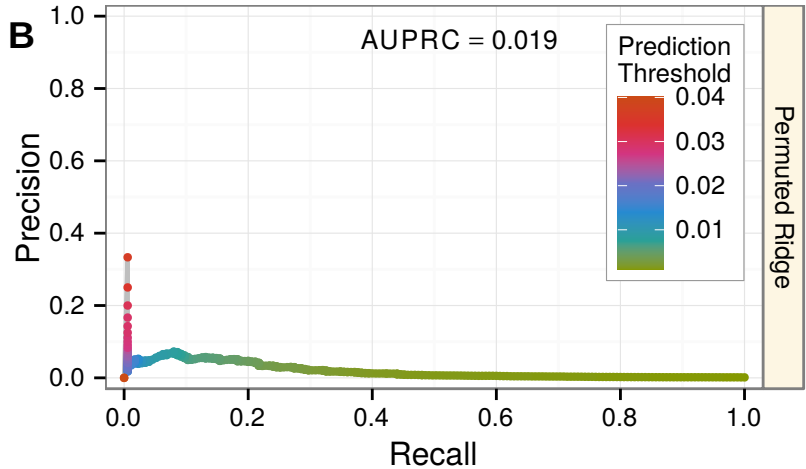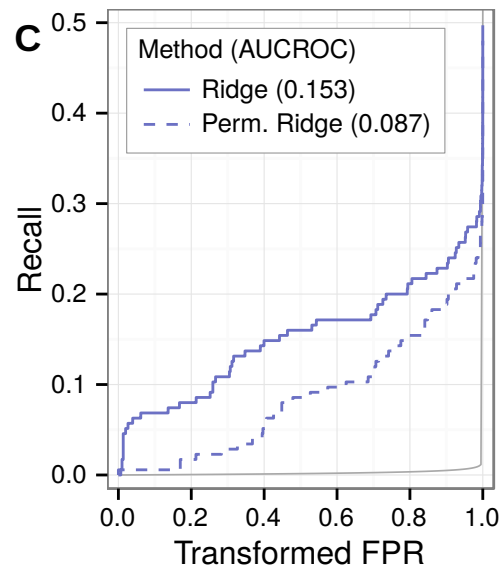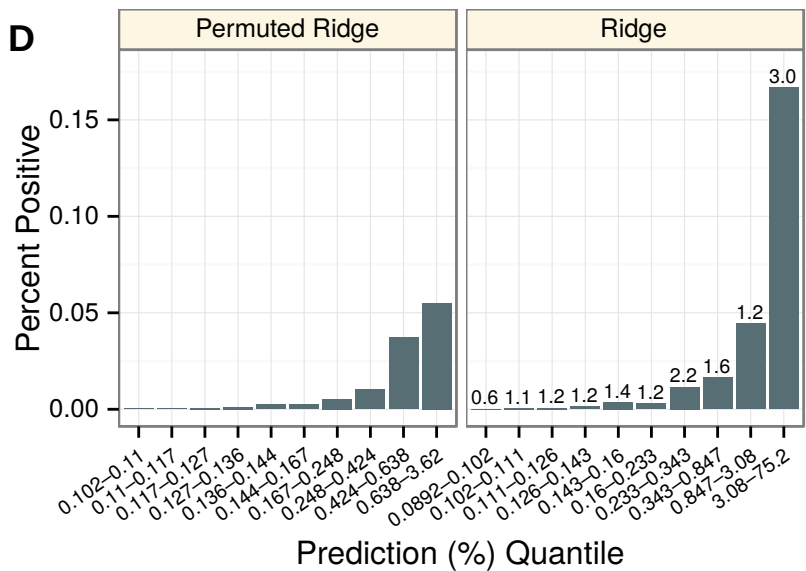

Supplement: S4 Fig — Testing performance is contrasted between ridge models for the permuted-network and unpermuted-network. A) Testing and training ROC curves for the permuted-network model. B) Testing precision-recall curve for the permuted-network model. C) Testing CROC curves for the permuted-network and unpermuted-network models. The FPR has been scaled to focus on the first 1% placing greater emphasis on top predictions. While both models vastly outperform random (grey line), the unpermuted-network model provides far superior top predictions. D) For both networks, gene-disease pairs were stratified by deciles of the predicted probabilities for positives. The x-axis labels show the predicted probabilities (as percentages) composing each decile. For each strata, the percent of positive pairs (precision) is plotted. The fold change over permuted is denoted for the unpermuted deciles. (PDF) [file pcbi.1004259.s004.pdf]

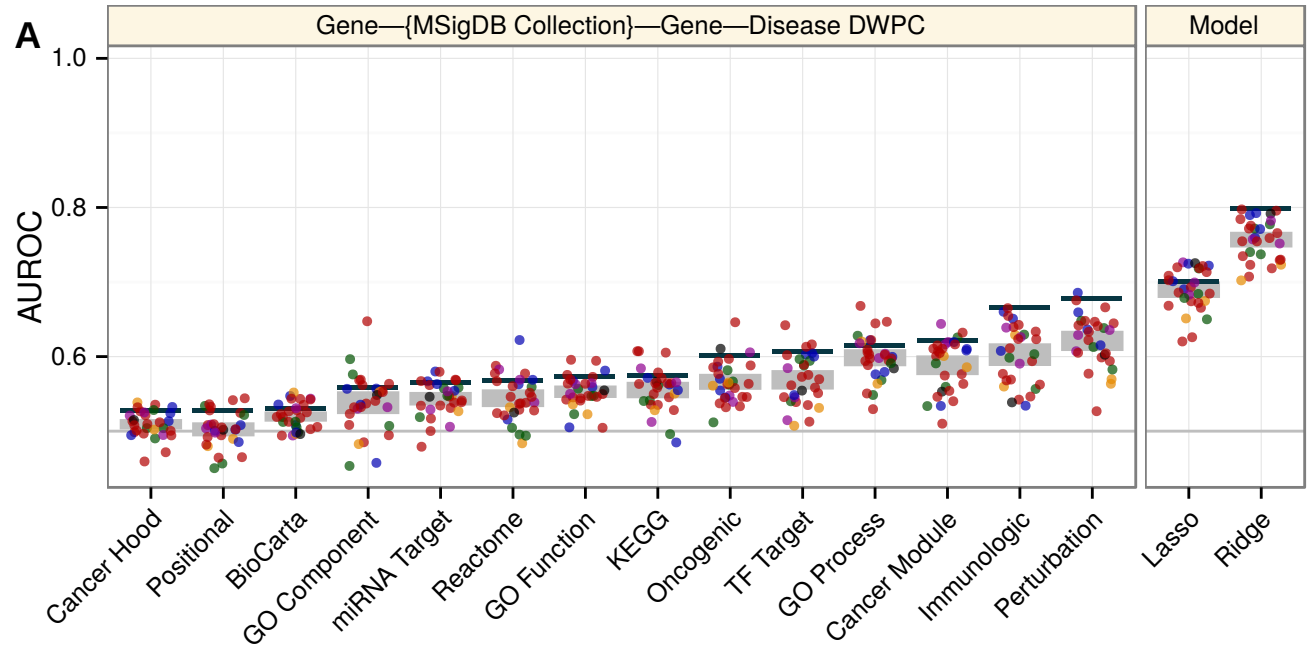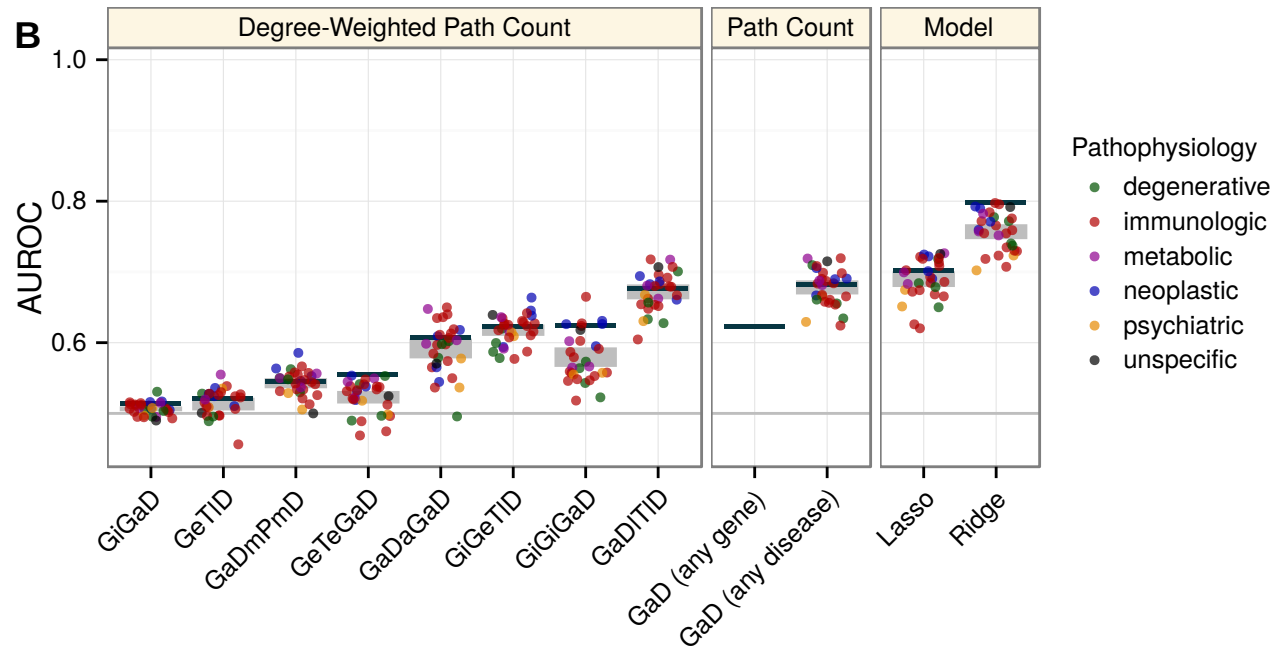

Supplement: S5 Fig — Disease, feature, and model-specific AUROCs were calculated separately for each of 5 permuted networks and averaged. The figure is analogous to Fig 5, except all measures refer to permuted-network performance. Disease-specific performance tends towards the mean, as disease-specific information has been altered by permutation. For features ending with an association (GaD) metaedge, global performance exceeds disease-specific performance. These features capture disease polygenicity, which improves the ranking of gene-disease pairs only if multiple diseases are included. Performance of the lasso model is affected, since the signals become too weak and few features survive regularization. (PDF) [file pcbi.1004259.s005.pdf]

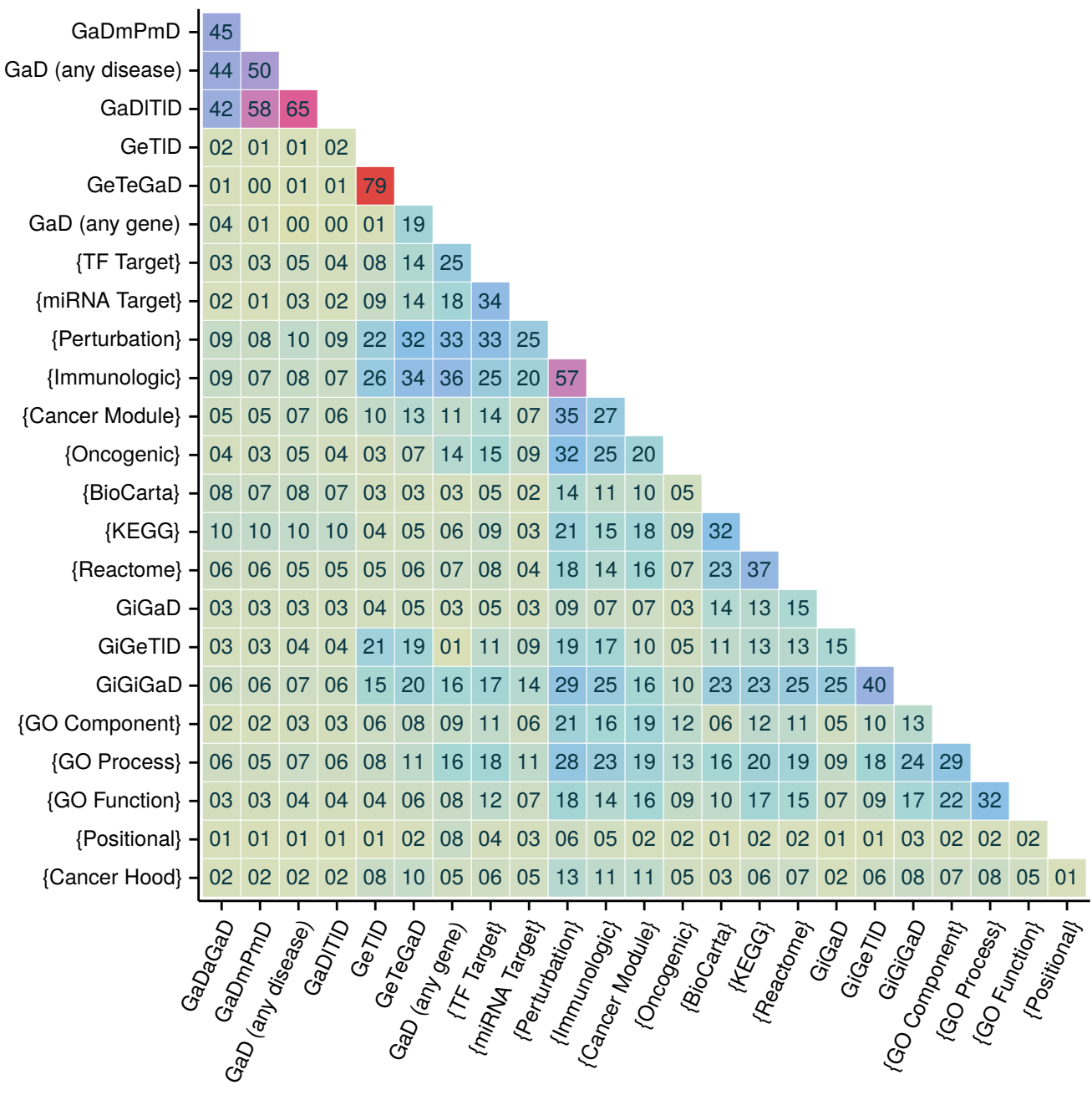

Supplement: S6 Fig — Pearson’s correlation coefficients (shown by color and as a percent) were calculated for all pairwise feature combinations. Features were ordered using Ward’s hierarchical clustering. Moderate collinearity is pervasive across features. The four pleiotropy-focused features form a tight cluster (top left). Perturbations and Immunologic signatures are correlated with many other features, including several other MSigDB features. (PDF) [file pcbi.1004259.s006.pdf]

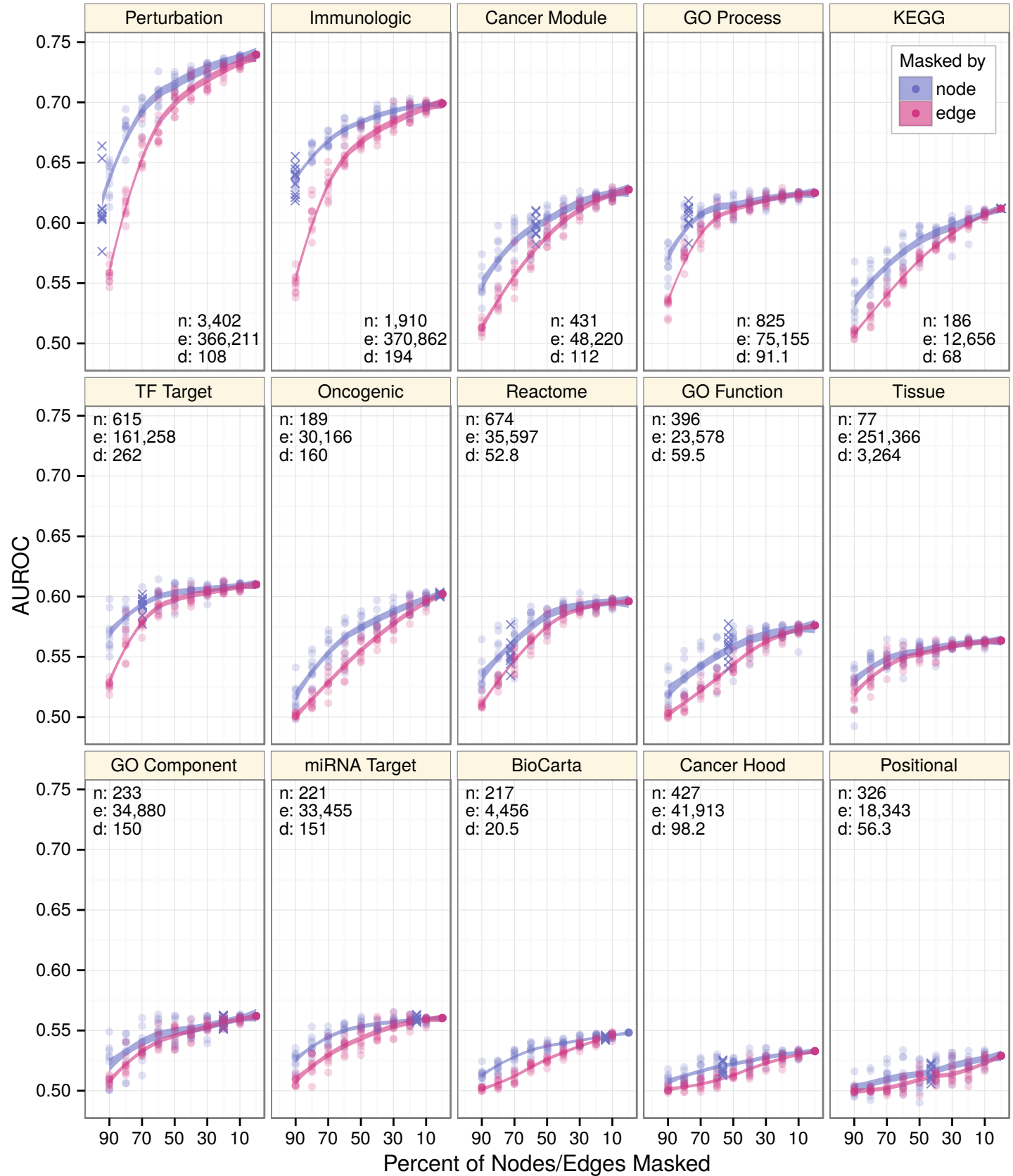

Supplement: S7 Fig — The effect of sparsity on feature performance is displayed for each gene set. The provided gene set information refers to node number (n), edge number (e), and mean degree (d). Crosses show performance for each gene after being subsampled to 186 nodes, corresponding to the number of pathways in KEGG—the MSigDB gene set in our network with the fewest nodes. The 95% loess confidence bands show expected performance across the entire range of node and edge masking percentages. (PDF) [file pcbi.1004259.s007.pdf]

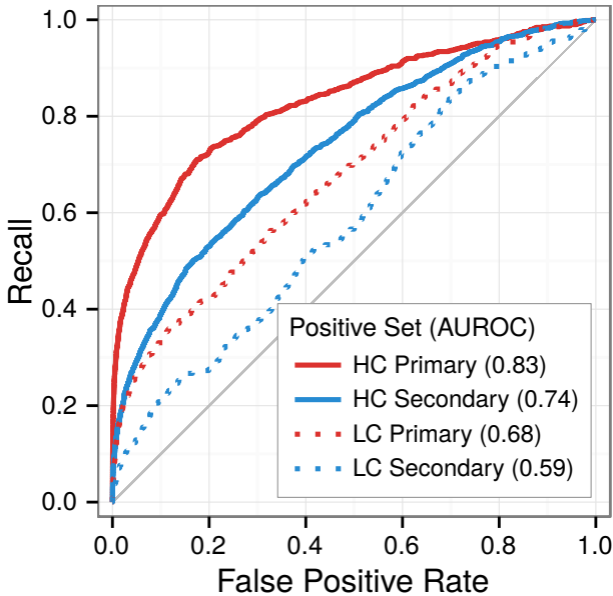

Supplement: S8 Fig — Keeping unassociated gene-disease pairs as negatives, ROC curves were calculated separately for each category of association as positives. Predictions from the complete-network ridge model were used as the classifier. For both high and low-confidence associations, primary gene annotations received higher predictions than secondary gene annotations. High-confidence associations received considerably higher predictions than low-confidence associations suggesting a high frequency of false positives amongst low-confidence associations. (PDF) [file pcbi.1004259.s008.pdf]

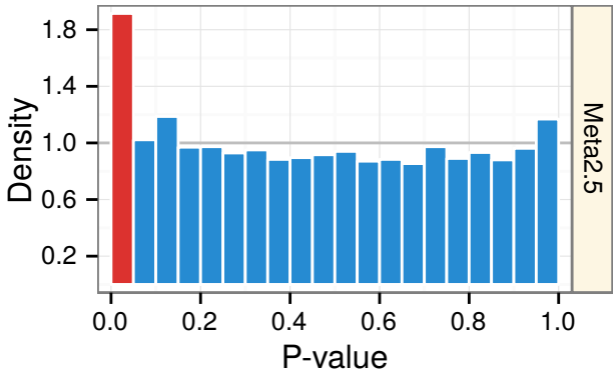

Supplement: S9 Fig — The histogram of genewise p-values from Meta2.5, a meta-analysis of multiple sclerosis GWAS preceding the WTCCC2 study. If no associations are present, uniformly distributed p-values (grey line) would be expected. Instead, we observed an excess of nominally significant genes (p ≤ 0.05, red) indicating a set of genes likely enriched for true associations. (PDF) [file pcbi.1004259.s009.pdf]

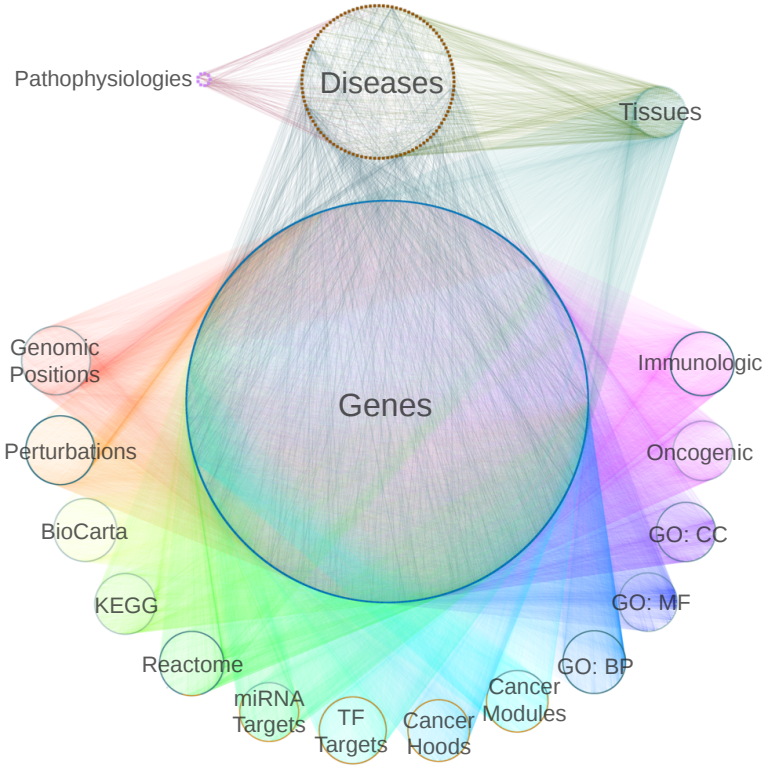

Supplement: S13 Data — PDF formatted versions of the figures. (ZIP) [file pcbi.1004259.s025.zip › Fig1.pdf]

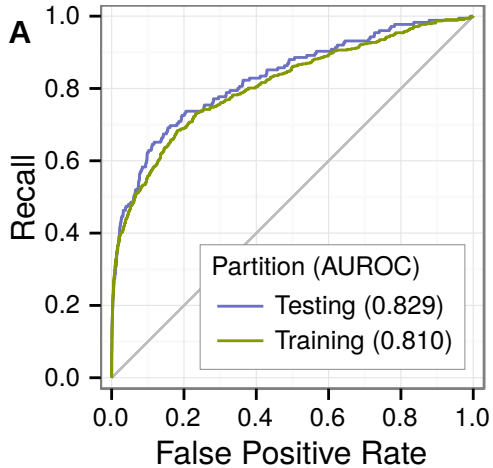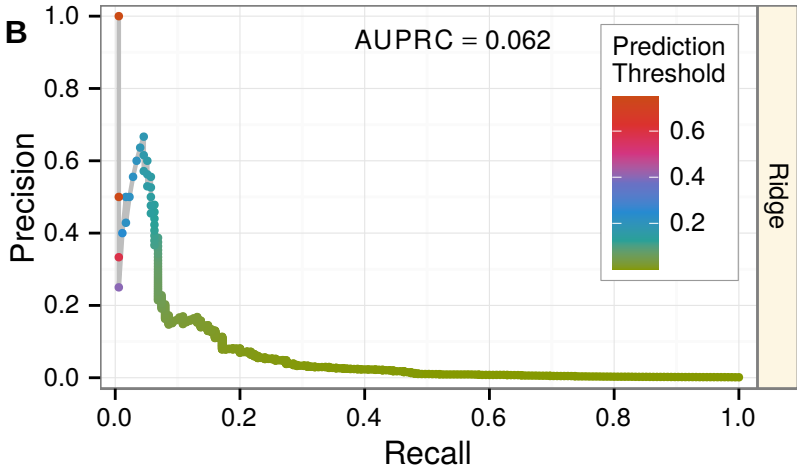

Supplement: S13 Data — PDF formatted versions of the figures. (ZIP) [file pcbi.1004259.s025.zip › Fig3.pdf]

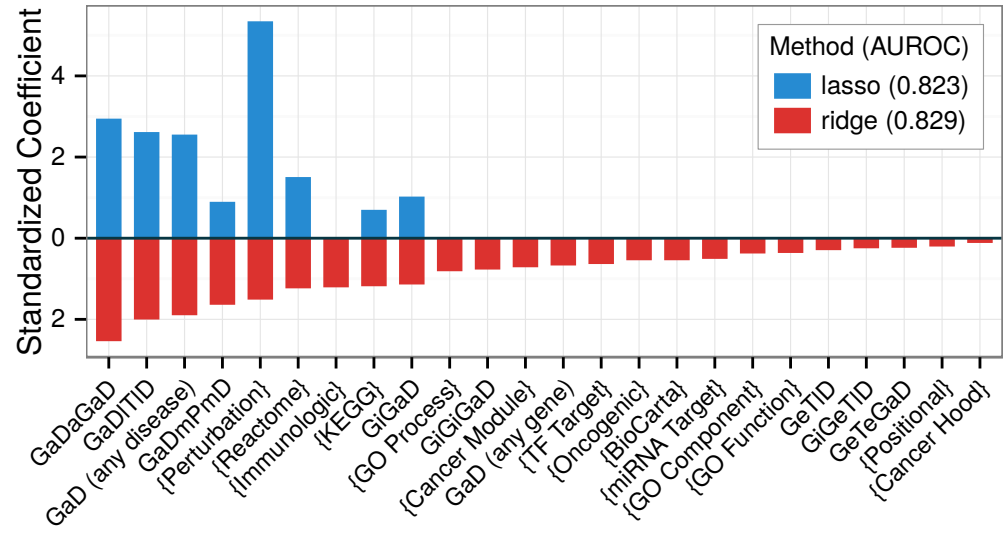

Supplement: S13 Data — PDF formatted versions of the figures. (ZIP) [file pcbi.1004259.s025.zip › Fig4.pdf]

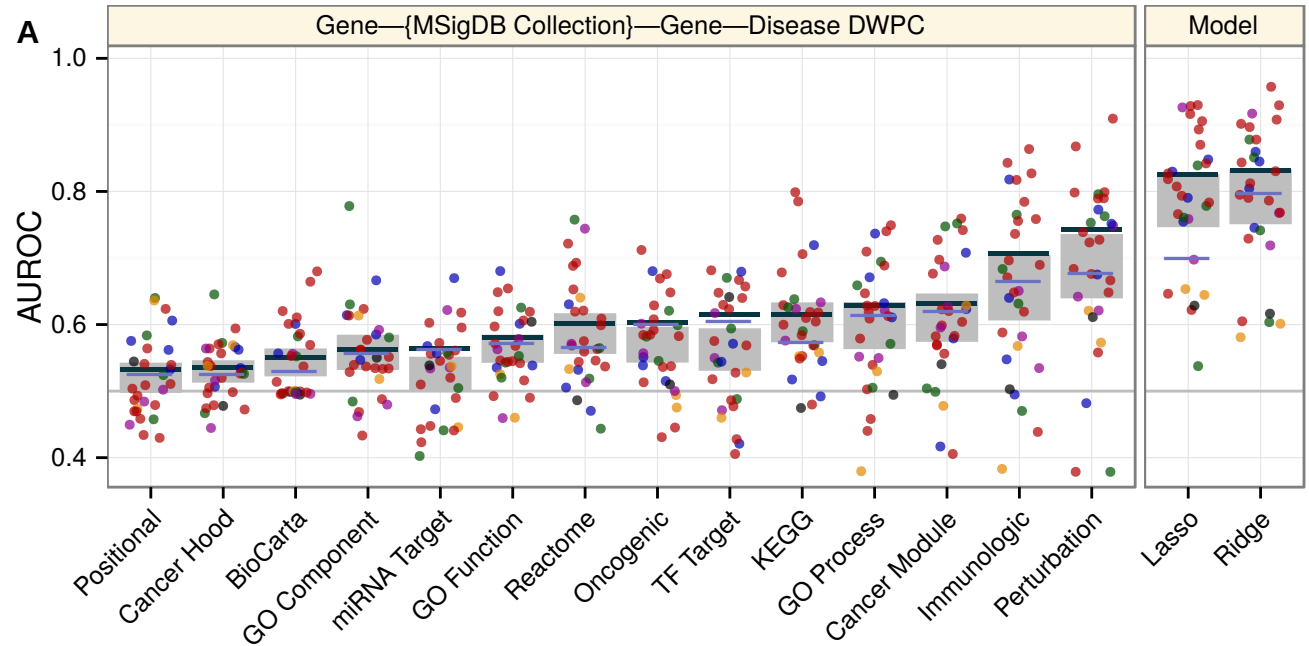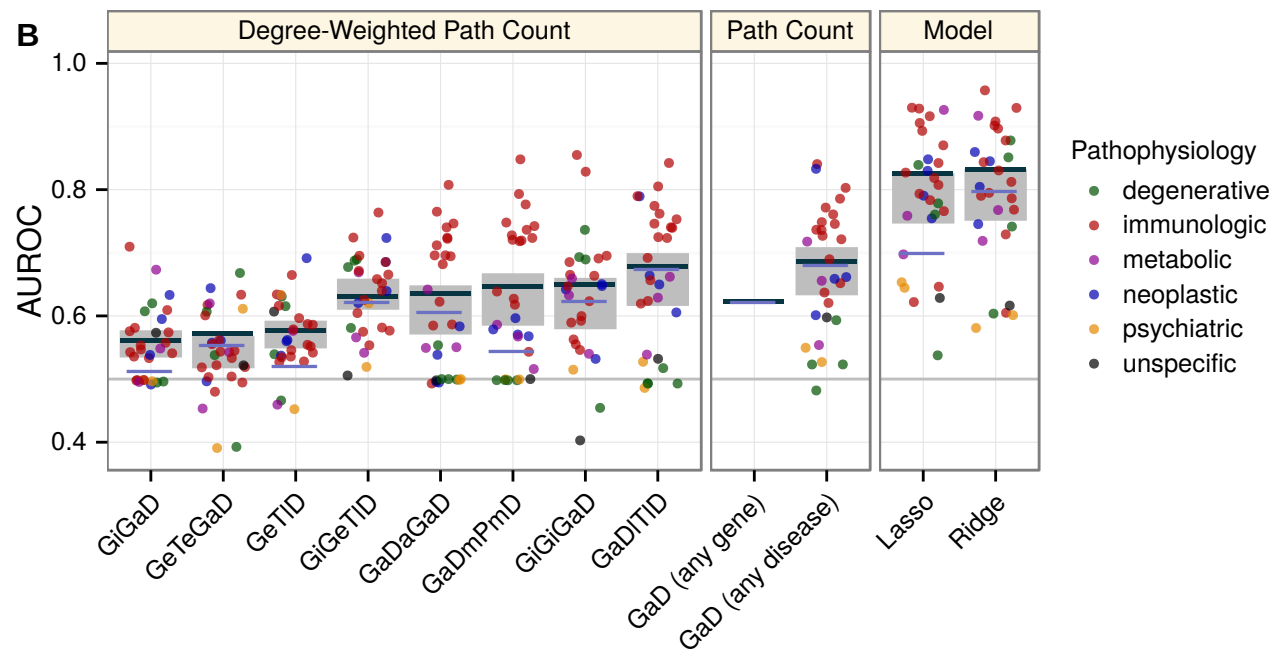

Supplement: S13 Data — PDF formatted versions of the figures. (ZIP) [file pcbi.1004259.s025.zip › Fig5.pdf]

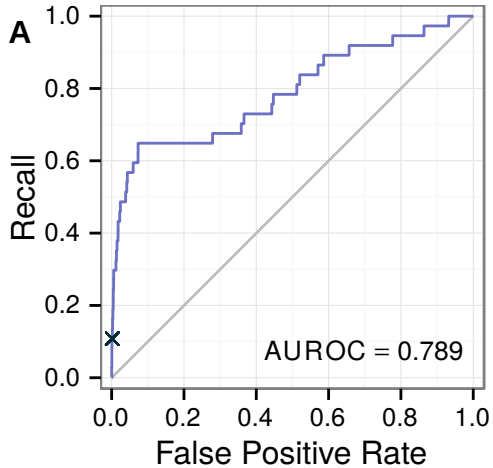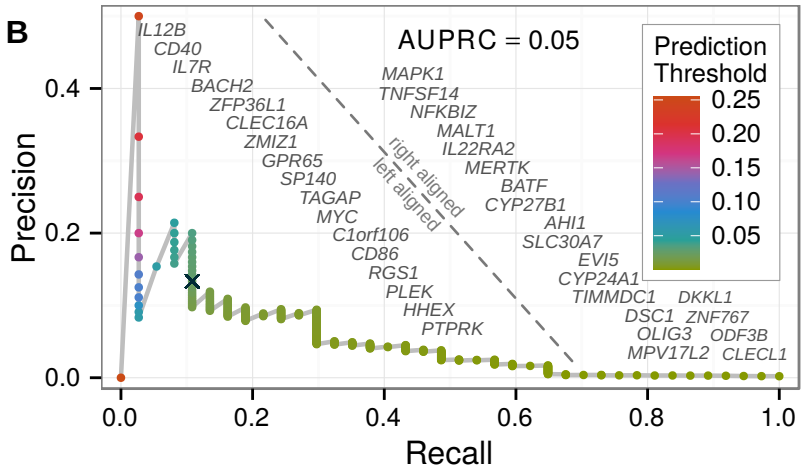

Supplement: S13 Data — PDF formatted versions of the figures. (ZIP) [file pcbi.1004259.s025.zip › Fig6.pdf]

# High Confidence Association:

Primary Gene: HLA-DRB1

Secondary Genes: HLA-DRA, C6orf10

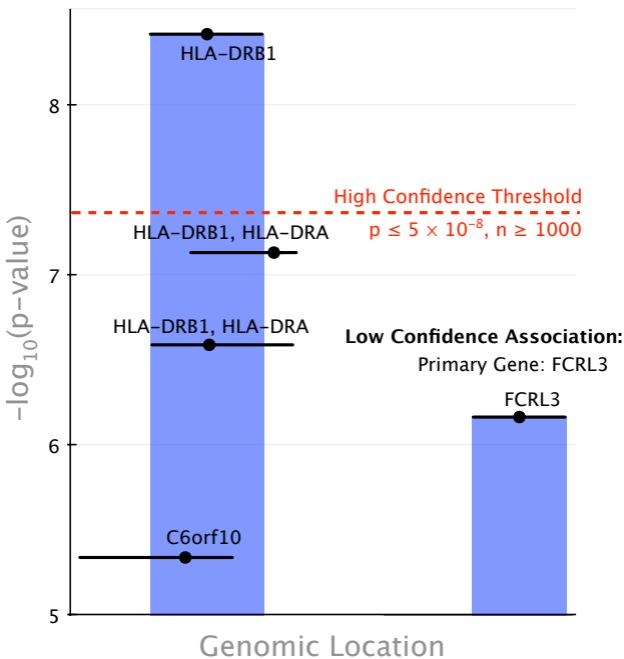

Supplement: S13 Data — PDF formatted versions of the figures. (ZIP) [file pcbi.1004259.s025.zip › Fig_S1.pdf]
