# Supplementary material for: Heterogeneous Network Edge Prediction: A Data Integration Approach to Prioritize Disease-Associated Genes
Source: PLoS Comput Biol. 2015 Jul 9;11(7):e1004259. doi: 10.1371/journal.pcbi.1004259 (PMC4497619; doi:10.1371/journal.pcbi.1004259)
Supplement: S1 Table — The 24 features computed for each gene-disease pair and the aspect of network topology described. (PDF) [file pcbi.1004259.s010.pdf]

**Path Count**	**Measures the number of ...**
GaD (any disease)	diseases that the source gene is associated with, ignoring the association with the target disease if present.
GaD (any gene)	genes that the target disease is associated with, ignoring the association with the source gene if present.
**DWPC**	**Measures the extent that ...**
GeTlD	the source gene is expressed in tissues affected by target disease.
GiGaD	genes associated with the target disease interact with the source gene.
GiGiGaD	genes associated with the target disease interact with genes that interact with the source gene.
GaDaGaD	genes associated with the same diseases as the source gene are associated with the target disease.
GaDmPmD	diseases with the same pathophysiology as the target disease are associated with the source gene.
GaDlTlD	diseases affecting the same tissues as the target disease are associated with the source gene.
GeTeGaD	genes expressed in the same tissues as the source gene are associated with the target disease.
GiGeTlD	genes interacting with the source gene are expressed in tissues that are affected by the target disease.
{Positional}	genes located in the same cytogenetic band as the source gene are associated with the target disease.
{Perturbation}	genes belonging to the same perterbation signatures as the source gene are associated with the target disease.
{BioCarta}	genes involved in the same BioCarta pathways as the source gene are associated with the target disease.
{KEGG}	genes involved in the same KEGG pathways as the source gene are associated with the target disease.
{Reactome}	genes involved in the same Reactome pathways as the source gene are associated with the target disease.
{miRNA Target}	genes sharing 3'-UTR microRNA binding motifs with the source gene are associated with the target disease.
{TF Target}	genes sharing transcription factor binding sites with the source gene are associated with the target disease.
{Cancer Hood}	genes present in the same expression neighborhoods of cancer-related genes as the source gene are associated with the target disease.
{Cancer Module}	genes belonging to the same cancer modules as the source gene are associated with the target disease.
{GO Process}	genes participating in the same GO Biological Processes as the source gene are associated with the target disease.
{GO Component}	genes belonging to the same GO Cellular Components as the source gene are associated with the target disease.
{GO Function}	genes contributing to the same GO Molecular Functions as the source gene are associated with the target disease.
{Oncogenic}	genes belonging to the same cancer-dysregulated cellular pathways as the source gene are associated with the target disease.
{Immunologic}	genes belonging to the same immunologic signatures as the source gene are associated with the target disease.
