# Supplementary material for: Heterogeneous Network Edge Prediction: A Data Integration Approach to Prioritize Disease-Associated Genes
Source: PLoS Comput Biol. 2015 Jul 9;11(7):e1004259. doi: 10.1371/journal.pcbi.1004259 (PMC4497619; doi:10.1371/journal.pcbi.1004259)
Supplement: S2 Table — Ten features (bold) showed a significant (p < 0.05, one-sided DeLong test) decrease in performance. (PDF) [file pcbi.1004259.s011.pdf]

| Feature | AUROC | p-AUROC | p-value |
|---|---|---|---|
| **GaDmPmD** | 0.643 | 0.547 | $1.6 \times 10^{-20}$ |
| **GiGaD** | 0.558 | 0.514 | $2.1 \times 10^{-9}$ |
| **{Perturbation}** | 0.740 | 0.667 | $2.3 \times 10^{-7}$ |
| **GeTlD** | 0.573 | 0.518 | $1.8 \times 10^{-5}$ |
| **{KEGG}** | 0.613 | 0.566 | $5.6 \times 10^{-5}$ |
| **GaDaGaD** | 0.633 | 0.592 | $1.5 \times 10^{-4}$ |
| **{BioCarta}** | 0.548 | 0.526 | 0.001 |
| **{Reactome}** | 0.599 | 0.562 | 0.002 |
| **{Immunologic}** | 0.703 | 0.665 | 0.006 |
| **GiGiGaD** | 0.646 | 0.621 | 0.05 |
| {Cancer Module} | 0.629 | 0.612 | 0.11 |
| GeTeGaD | 0.570 | 0.554 | 0.14 |
| {TF Target} | 0.612 | 0.596 | 0.16 |
| {GO Component} | 0.560 | 0.547 | 0.17 |
| {Positional} | 0.529 | 0.520 | 0.19 |
| GiGeTlD | 0.628 | 0.616 | 0.20 |
| {GO Process} | 0.626 | 0.617 | 0.26 |
| GaD (any disease) | 0.683 | 0.676 | 0.30 |
| {GO Function} | 0.577 | 0.571 | 0.31 |
| {Cancer Hood} | 0.533 | 0.532 | 0.45 |
| GaD (any gene) | 0.620 | 0.620 | 0.49 |
| GaDlTlD | 0.674 | 0.674 | 0.49 |
| {Oncogenic} | 0.601 | 0.604 | 0.60 |
| {miRNA Target} | 0.562 | 0.569 | 0.72 |
