# Supplementary material for: Heterogeneous Network Edge Prediction: A Data Integration Approach to Prioritize Disease-Associated Genes
Source: PLoS Comput Biol. 2015 Jul 9;11(7):e1004259. doi: 10.1371/journal.pcbi.1004259 (PMC4497619; doi:10.1371/journal.pcbi.1004259)
Supplement: S3 Table — The upper section details the high-performing network prediction threshold. The lower section details the hypergeometric test for overrepresentation of validating genes. (PDF) [file pcbi.1004259.s012.pdf]

	Value
**Prediction Threshold**	0.024
**False Positive Rate**	0.001
**Recall**	0.108
**Precision**	0.133
**Lift**	68.4
**Novel & Meta2.5-nominal Total**	1211
**Novel & $\geq$ Prediction Threshold**	19
**Discovered**	4
**Bonferroni Cutoff**	0.0125
**Discovered <Bonferroni**	3
**Total <Bonferroni**	199
**Replication *p*-value**	0.015
