# Supplementary material for: Heterogeneous Network Edge Prediction: A Data Integration Approach to Prioritize Disease-Associated Genes
Source: PLoS Comput Biol. 2015 Jul 9;11(7):e1004259. doi: 10.1371/journal.pcbi.1004259 (PMC4497619; doi:10.1371/journal.pcbi.1004259)
Supplement: S13 Data — PDF formatted versions of the figures. (ZIP) [file pcbi.1004259.s025.zip › Fig2.pdf]

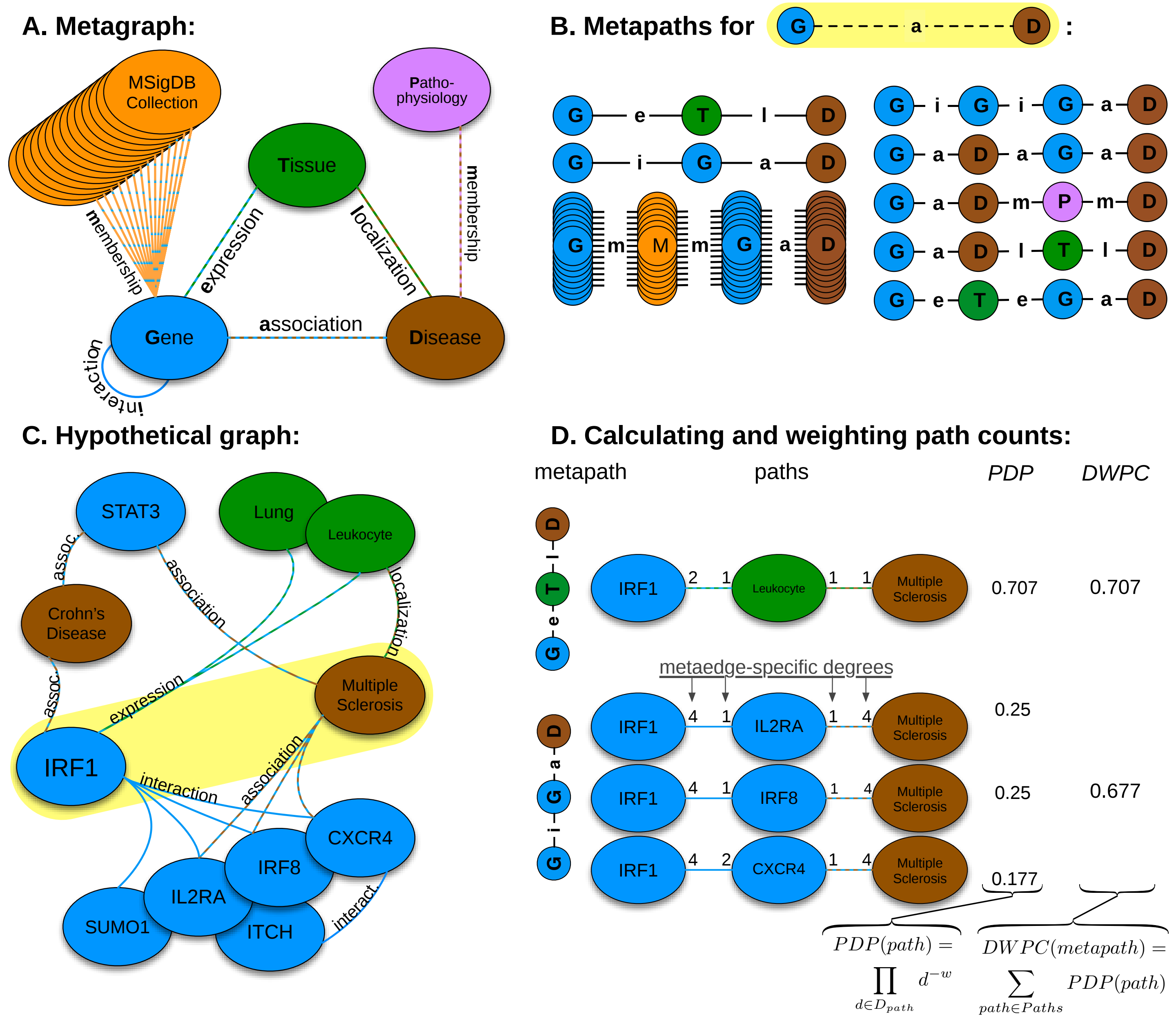
A. Metagraph:
MSigDB Collection
Patho-physiology
Tissue
membership
expression
localization
membership
Gene
association
Disease
interaction
B. Metapaths for G a D :
G e T l D
G i G a D
G m M m G a D
G i G i G a D
G a D a G a D
G a D m P m D
G a D l T l D
G e T e G a D
C. Hypothetical graph:
STAT3
Lung
Leukocyte
assoc.
association
localization
Crohn's Disease
Multiple Sclerosis
assoc.
expression
IRF1
interaction
association
CXCR4
IRF8
IL2RA
SUMO1
ITCH
interact.
D. Calculating and weighting path counts:
metapath
paths
PDP
DWPC
G e T l D
IRF1 2 1 Leukocyte 1 1 Multiple Sclerosis
0.707
0.707
metaedge-specific degrees
G i G a D
IRF1 4 1 IL2RA 1 4 Multiple Sclerosis
0.25
IRF1 4 1 IRF8 1 4 Multiple Sclerosis
0.25
0.677
IRF1 4 2 CXCR4 1 4 Multiple Sclerosis
0.177
$PDP(path) = \prod_{d \in D_{path}} d^{-w}$
$DWPC(metapath) = \sum_{path \in Paths} PDP(path)$
